# GENDER DIFFERENCES IN THE PREVALENCE AND INCIDENCE OF COGNITIVE IMPAIRMENT: DOES IMMIGRATION MATTER?

**DOI:** 10.1093/geroni/igz038.3003

**Published:** 2019-11-08

**Authors:** Fengyan Tang, Fengyan Tang, Ke Li, Iris Chi, XinQi Dong

**Affiliations:** 1 University of Pittsburgh, Pittsburgh, Pennsylvania, United States; 2 University of Southern California, Los Angeles, California, United States; 3 Rutgers University, The State University of New Jersey, New Brunswick, New Jersey, United States

## Abstract

Using two waves of the PINE data, this study examined gender difference in the associations between immigration-related factors and cognitive impairment (CI). CI was assessed by the Chinese Mini-Mental State Examination (C-MMSE). CI prevalence was determined by C-MMSE < 18 at baseline; incidence was the percentage of the respondents whose C-MMSE > 18 at baseline but dropped below 18 at Wave 2. We found 7.62% CI prevalence and 5.12% incidence rate. Women were more likely than men to have CI, consisting of 77.06% and 75.20% among persons with CI at two time points. Older Chinese women were generally disadvantaged in cognition and overall health compared with older men. Yet immigration experience does not link to CI for both men and women after controlling the well-established effects of age and education. Future research needs to investigate what biological and contextual factors earlier in life are predictive of late-life CI risk.

